# Prognostic Factors Research in Breast Cancer Patients: New Paths

**DOI:** 10.3390/cancers14040971

**Published:** 2022-02-15

**Authors:** Tommaso Susini, Nicoletta Biglia, Valentina Elisabetta Bounous

**Affiliations:** 1 Breast Unit, Gynecology Section, Department of Health Sciences, University of Florence, 50121 Firenze, Italy; 2 Academic Division of Obstetrics and Gynecology, Mauriziano Umberto 1st Hospital, School of Medicine, University of Torino, 10128 Torino, Italy; nicoletta.biglia@unito.it (N.B.); valentinabounous@hotmail.com (V.E.B.)

Breast cancer is the most frequent tumor among women worldwide [1]. Its incidence is increasing at all ages, while mortality is decreasing due to improvements in screening programs and in treatment. 

Breast cancer is a heterogeneous disease, so treatment and prognosis depend on tumor subtype, grade, lymph node status and stage of disease; however even patients with similar prognostic features may have different clinical outcome. For this reason, great effort must made in research focusing on new prognostic factors.

This Special Issue aims to present, through the publication of 10 original articles, new approaches to individualize the prognosis and treatment of breast cancer patients. 

The identification of new prognostic factors is crucial for improvement in breast cancer care, and both molecular and immunohistochemical studies are important. It is important to also consider the sustainability of costs, because only methods affordable for the majority of countries will have wide enough distribution to impact the clinical outcome of women with breast cancer worldwide.

Regarding studies employing molecular techniques, three studies in this Special Issue focus on new prognostic factors, and one study analyzed gene expression profile changes during breast cancer metastasis. 

Among new prognostic factors, circulating tumor cells (CTCs), both in early-stage and metastatic breast cancer patients, are useful in discriminating cases at high risk of relapse [2]. In the study by Rossi T. et al, the molecular aspect of CTCs was evaluated on 11 early-stage breast cancer patients, focusing on copy number alterations and exploiting a single-CTC next-generation sequencing approach [3]. Furthermore, resistance to apoptosis is a hallmark of cancer, and the inhibitors of apoptosis proteins (IAP family) play a key role in regulating cell death [4]. The most potent protein of this family is the X-linked inhibitor of apoptosis protein (XIAP), which is important during the mammary gland remodeling process, in particular after each menstrual cycle and during the transformation of breast ducts post lactation [5]. XIAP expression confers resistance to chemotherapy and tumor aggressiveness. Devi et al. confirmed, in a large-scale study on 2341 breast samples, the poorer prognosis and resistance to chemotherapy associated with XIAP expression [6]. Moreover, another family of protein-coding genes, “Like-Smith” (LSM), plays an important role in the progression of several malignancies, although its role in breast cancer is unclear. In the study by Ta et al., the clinical relevance of LSM family genes were evaluated through multiple analyses based on 3593 patients with breast cancer and their mRNA expression. In their findings, LSM4 expression was associated with poor survival outcomes [7]. 

Several genes associated with distant organ-specific metastasis have been identified through the literature [8]. To improve the understanding of how the gene expression profile changes during the breast cancer metastasis process, Maiti et al. compared the primary tumor and distant metastatic tumor gene expression in animal models [9], finding that mediators encoding genes with prognostic and predictive values may be clinically useful for breast cancer spine, bone, and lung metastasis, particularly in more aggressive subtypes such as triple-negative and HER2+ breast cancer.

Two studies of this Special Issue evaluated new prognostic factors for breast cancer by immunohistochemistry.

FGD3 (Facio-Genital Dysplasia 3) gene expression is associated with improved outcomes since it inhibits cell migration. In the study by Susini et al., the prognostic role of FGD3 was evaluated in a series of 401 women with invasive breast cancer by immunohistochemistry. Patients with high FGD3 expression showed a significantly better disease-free survival and overall survival. The prognostic value of FGD3 was stronger than all classic pathologic features, except for stage. The possibility to assess FGD3 expression by a simple and cheap technique such as immunohistochemistry may enhance the spread of its use in clinical practice [10].

Therapies targeting nuclear receptors, such as the estrogen and the progesterone receptors, are established treatment options for breast cancer. Besides the well-known nuclear receptors, others, including the retinoid X receptor (RXR), thyroid hormone receptors and vitamin D receptor, play a significant role in the pathophysiology of breast cancer. The study by Zheni et al. aimed to assess the prognostic value of nuclear versus cytoplasmic retinoid X receptor α (RXRα) expression by immunohistochemistry in 319 breast cancer patients [11]. In their findings, the expression of cytoplasmic RXRα is correlated with a more aggressive disease, whereas nuclear RXRα expression appears to be a protective factor. 

The knowledge of new prognostic factors over time has led to improvements in treatments, for both primary and metastatic breast cancer, and as a consequence, to improved survival. The estimated five-year survival is over 85% in developed countries; however, in developing countries, the five-year survival is lower, ranging from 64.8 to 68.7% [12]. 

Many clinical tools for predicting the survival of breast cancer patients have been developed over the years. However, most of them were developed in Western countries and do not perform well for other ethnicities, such as the Asian population. In this Special Issue, we included a study from Thailand proposing a new model for survival predictions, using modern statistical methods that allow a more accurate estimation of the baseline survival [13]. 

Clinical outcome in the metastatic setting was analyzed in another study of this Special Issue. De novo metastatic breast cancer (dnMBC) is breast cancer with metastasis at the time of initial diagnosis and seems to have a better overall survival compared with recurrent metastatic breast cancer (rMBC) [14]. Iwase et al. performed a retrospective study on 1981 breast cancer patients, to assess the change in overall survival for patients with dnMBC over time. They found a significant improvement in overall survival over time for the estrogen receptor-positive/HER2+ subtype, suggesting the contribution of HER2-targeted therapy to survival. In addition, having non-inflammatory breast cancer, hormone-positive disease, HER2+ disease and metastasis to a single organ conferred a longer overall survival [15].

As regards adjuvant treatments, hormone therapy is the standard for hormone-dependent cancer and chemotherapy is widely used for triple-negative tumors and high-risk patients [16].

Since the early 2000s, the recombinant humanized monoclonal antibody trastuzumab has been used to treat patients with HER2-positive tumors, being highly effective, showing a favorable benefit/risk profile, leading to significant gains in overall and disease-free survival [17]. Even if trastuzumab is routinely administrated to patients with HER2+ cancer, its use in small node-negative HER2+ breast cancers is still controversial. The decision to prescribe it in this subgroup of patients must balance the known toxicities of this treatment and the uncertain benefits that can be achieved [18]. A multicenter study included in this Special Issue evaluated the effect of trastuzumab in the early stages of breast cancer (pT1mic/pT1a pN0/1mi) in terms of disease recurrence and analyzed the factors that most affect the prognosis of small HER2+ tumors [19]. 

Furthermore, among patients with breast cancer, comorbidities in general, and specifically cardiovascular diseases, chronic obstructive pulmonary disease, diabetes, and venous thromboembolism, negatively affect overall survival [20]. A study in this Special Issue evaluated whether the severity of smoking-related chronic obstructive pulmonary disease was a significant prognostic factor for overall survival in breast cancer patients receiving standard treatments [21]. 

In the era of precision medicine, increasing efforts must be directed to finding new prognostic biomarkers for breast cancer, allowing for a more personalized approach, to develop new tailored treatments and ultimately to further improve the outcome of our patients.

The readers of this Special Issue will receive an important update on current knowledge in breast cancer research, and especially in the field of prognostic factors.

## References

[1] World Health Organization (2021). Breast Cancer.

[2] Pantel K., Speicher M.R. (2016). The biology of circulating tumor cells. Oncogene.

[3] Rossi T., Gallerani G., Angeli D., Cocchi C., Bandini E., Fici P., Gaudio M., Martinelli G., Rocca A., Maltoni R. (2020). Single-Cell NGS-Based Analysis of Copy Number Alterations Reveals New Insights in Circulating Tumor Cells Persistence in Early-Stage Breast Cancer. Cancers.

[4] Jost P.J., Vucic D. (2020). Regulation of Cell Death and Immunity by XIAP. Cold Spring Harb. Perspect. Biol..

[5] Owens T.W., Foster F.M., Tanianis-Hughes J., Cheung J.Y., Brackenbury L., Streuli C.H. (2010). Analysis of inhibitor of apoptosis protein family expression during mammary gland development. BMC Dev. Biol..

[6] Devi G.R., Finetti P., Morse M.A., Lee S., de Nonneville A., Van Laere S., Troy J., Geradts J., McCall S., Bertucci F. (2021). Expression of X-Linked Inhibitor of Apoptosis Protein (XIAP) in Breast Cancer Is Associated with Shorter Survival and Resistance to Chemotherapy. Cancers.

[7] Ta H.D.K., Wang W.-J., Phan N.N., An Ton N.T., Anuraga G., Ku S.-C., Wu Y.-F., Wang C.-Y., Lee K.-H. (2021). Potential Therapeutic and Prognostic Values of LSM Family Genes in Breast Cancer. Cancers.

[8] Minn A.J., Kang Y., Serganova I., Gupta G.P., Giri D.D., Doubrovin M., Ponomarev V., Gerald W.L., Blasberg R., Massague J. (2005). Distinct organ-specific metastatic potential of individual breast cancer cells and primary tumors. J. Clin. Investig..

[9] Maiti A., Okano I., Oshi M., Okano M., Tian W., Kawaguchi T., Katsuta E., Takabe K., Yan L., Patnaik S.K. (2021). Altered Expression of Secreted Mediator Genes That Mediate Aggressive Breast Cancer Metastasis to Distant Organs. Cancers.

[10] Susini T., Saccardin G., Renda I., Giani M., Tartarotti E., Nori J., Vanzi E., Pasqualini E., Bianchi S. (2021). Immunohistochemical Evaluation of FGD3 Expression: A New Strong Prognostic Factor in Invasive Breast Cancer. Cancers.

[11] Zati Zehni A., Batz F., Cavaillès V., Sixou S., Kaltofen T., Keckstein S., Heidegger H.H., Ditsch N., Mahner S., Jeschke U. (2021). Cytoplasmic Localization of RXRα Determines Outcome in Breast Cancer. Cancers.

[12] Allemani C., Matsuda T., Di Carlo V., Harewood R., Matz M., Nikšić M., Bonaventure A., Valkov M., Johnson C.J., Estève J. (2018). Global Surveillance of Trends in Cancer Survival 2000-14 (CONCORD-3): Analysis of Individual Records for 37,513,025 Patients Diagnosed with One of 18 Cancers from 322 Population-Based Registries in 71 Countries. Lancet Lond. Engl..

[13] Pongnikorn D., Phinyo P., Patumanond J., Daoprasert K., Phothong P., Siribumrungwong B. (2021). Individualized Prediction of Breast Cancer Survival Using Flexible Parametric Survival Modeling: Analysis of a Hospital-Based National Clinical Cancer Registry. Cancers.

[14] Tripathy D., Brufsky A., Cobleigh M., Jahanzeb M., Kaufman P.A., Mason G., O’Shaughnessy J., Rugo H.S., Swain S.M., Yardley D.A. (2020). De Novo Versus Recurrent HER2-Positive Metastatic Breast Cancer: Patient Characteristics, Treatment, and Survival from the SystHERs Registry. Oncologist.

[15] Iwase T., Shrimanker T.V., Rodriguez-Bautista R., Sahin O., James A., Wu J., Shen Y., Ueno N.T. (2021). Changes in Overall Survival over Time for Patients with de novo Metastatic Breast Cancer. Cancers.

[16] Burstein H.J., Curigliano G., Loibl S., Dubsky P., Gnant M., Poortmans P., Colleoni M., Denkert C., Piccart-Gebhart M., Regan M. (2019). Members of the St. Gallen International Consensus Panel on the Primary Therapy of Early Breast Cancer 2019. Estimating the benefits of therapy for early-stage breast cancer: The St. Gallen International Consensus Guidelines for the primary therapy of early breast cancer. Ann. Oncol..

[17] Viani G.A., Afonso S.L., Stefano E.J., De Fendi L.I., Soares F.V. (2007). Adjuvant trastuzumab in the treatment of her-2-positive early breast cancer: A meta-analysis of published randomized trials. BMC Cancer.

[18] National Comprehensive Cancer Network (2021). Breast Cancer (Version 1). https://www.nccn.org/professionals/physician_gls/pdf/breast.pdf.

[19] Villasco A., Actis S., Bounous V.E., Borella F., D’Alonzo M., Ponzone R., De Sanctis C., Benedetto C., Biglia N. (2021). The Role of Trastuzumab in Patients with HER2 Positive Small (pT1mi/a) Breast Cancers, a Multicenter Retrospective Study. Cancers.

[20] Janssen-Heijnen M.L., Maas H.A., Houterman S., Lemmens V.E., Rutten H.J., Coebergh J.W. (2007). Comorbidity in older surgical cancer patients: Influence on patient care and outcome. Eur. J. Cancer.

[21] Zhang J.-Q., Cheng T.-M., Lin W.-C., Chiu K.-C., Wu S.-Y. (2021). Impact of Smoking-Related Chronic Obstruction Pulmonary Disease on Mortality of Invasive Ductal Carcinoma Patients Receiving Standard Treatments: Propensity Score-Matched, Nationwide, Population-Based Cohort Study. Cancers.

